# High brightness and low operating voltage CsPbBr_3_ perovskite LEDs by single-source vapor deposition

**DOI:** 10.1038/s41598-024-54036-6

**Published:** 2024-02-09

**Authors:** Kuan-Chi Yeh, Chia-Hua Chan

**Affiliations:** Graduate Institute of Energy Engineering, National Central University, No. 300, Zhongda Rd., Zhongli Dist., Taoyuan City, 32001 Taiwan, ROC

**Keywords:** Inorganic LEDs, Materials for devices

## Abstract

In this work, we utilized CsPbBr_3_ powder as the precursor material for the single-source vapor deposition (SSVD) process to fabricate the CsPbBr_3_ emitting layer. Due to the high density of grain boundaries and defects in the thin films deposited in the initial stages, non-radiative recombination can occur, reducing the efficiency of perovskite light-emitting diodes (PeLED). To address this issue, we employed a thermal annealing process by subjecting the perovskite films to the appropriate annealing temperature, facilitating the coalescence and growth of different grains, improving lattice integrity, and thereby reducing the presence of defects and enhancing the photoluminescence performance of the films. Furthermore, in this study, we successfully fabricated simple-structured CsPbBr_3_ PeLED using thermally annealed CsPbBr_3_ films. Among these components, even without adding the electron and hole transport layers, the best-performing device achieved a maximum brightness of 14,079 cd/m^2^ at a driving voltage of only 2.92 V after annealing at 350 °C*;* the brightness is 16.8 times higher than that of CsPbBr_3_ PeLED without heat treatment, demonstrating outstanding light-emitting performance. The research results show that using SSVD to prepare CsPbBr_3_ PeLED has broad application potential, providing a simple process option for research on improving the performance of PeLED.

## Introduction

With the rapid advancements in technology, there is a growing demand for lighting, screens, TVs, smartphones, and wearable display devices in our daily lives. In recent years, the scientific community has discovered that perovskite is a semiconductor material with significant potential for a wide range of applications, and its exceptional performance has garnered substantial attention in various fields. Perovskite exhibits excellent light absorption coefficients^1,2^, allowing it to efficiently absorb photon energy and convert it into either photons or electrons. Perovskite boasts high external quantum efficiency, signifying its capacity to effectively convert absorbed photons or electrons into another form of energy. These superior optoelectronic properties make perovskite an ideal material for fabricating high-efficiency optoelectronic devices. Additionally, perovskite possesses outstanding carrier mobility^3,4^, which accelerates the movement of carriers within the material, further enhancing the performance of optoelectronic devices. Moreover, perovskite offers a tunable bandgap, allowing for the adjustment of its bandgap size by altering the material’s chemical composition or crystal structure to meet various optoelectronic device application requirements^5^. Due to the ease of acquiring and manufacturing perovskite materials, they can be fabricated in various forms, including thin films, crystals, or nanoparticles with different shapes and structures^6–9^. Currently, perovskite has found widespread applications in numerous fields, such as LED technology^10,11^, solar cells^12,13^, photodetectors^14,15^, and lasers^16,17^.

The chemical formula of perovskite is typically ABX_3_, where A represents a monovalent cation (such as CH_3_NH^3+^ or Cs^+^), B represents a divalent cation (such as Pb^2+^ or Sn^2+^), and X represents a halide anion (such as Cl^−^, Br^−^, and I^−^). By altering the composition of halide anions, the components and emission wavelength of perovskite can be adjusted^5,18,19^. Currently, the External Quantum Efficiency (EQE) of organic–inorganic metal halide perovskite LEDs has exceeded 20%^20^. However, these compositions contain organic cations such as methylamine (MA) or formamidine (FA), which have lower resistance to environmental moisture and oxygen. In contrast, the use of inorganic cesium ions (Cs) to replace organic cations, forming CsPbX_3_, is gaining significant attention in recent years due to its high efficiency and stability. Currently, there are two main categories of methods for preparing perovskite films. One category is the solution method, which includes techniques such as spin-coating^21^, spray-coating^22^, and inkjet printing^23,24^. The advantage of the solution method lies in its cost-effectiveness, ease of operation, and the ability to add ligands^25^ and adjust the ratio of halide ions. Therefore, it offers a simple and fast approach for producing films or perovskite light-emitting diodes (PeLED). For example, filling a solution into a pen-like device allows for rapidly completing the required film or PeLED process while maintaining good efficiency^26^. Although the solution method has already been used to create high-efficiency optoelectronic devices, it also has some limitations. For instance, the poor solubility of CsBr in dimethylformamide (DMF) and dimethyl sulfoxide (DMSO) hinders the increase in the concentration of precursor solutions^27^. Additionally, it can lead to defects in the film quality when excess ligands or solvents are removed^28^.

Another method for preparing perovskite films is the evaporation method, which can be divided into dual-source co-evaporation^29,30^, two-step evaporation^31,32^, and single-source vapor deposition SSVD^33,34^, among others. The advantage of the evaporation method is that it can produce highly stable, high-quality, and uniformly structured 3D grain surfaces, with significant potential for improving crystal quality. Currently, PeLED prepared using the evaporation method has achieved high-quality luminescent films with high uniformity^35^. That means component uniformity and consistency are ensured, whether in large panels or small components. Additionally, it is suitable for small-sized components, and by using high-precision metal masks, micron-sized patterning can be achieved, further expanding the range of applications^36^. This proves that the evaporation method can successfully prepare high-quality PeLED. Whether for large or small-sized components, it demonstrates excellent performance and versatility, making it one of the preferred methods for PeLED fabrication. Dual-source co-evaporation is the most commonly used method, which requires depositing two different source materials simultaneously from separate evaporation sources while precisely controlling the ratio and deposition rates of the two materials, making the process more challenging. To simplify the process, some studies use pre-synthesized perovskite powders for SSVD^37^, directly depositing them onto substrates. This process is relatively straightforward, and the resulting perovskite films exhibit good quality, low roughness, and fewer defects, making them competitive with films prepared using dual-source co-evaporation.

Currently, most PeLED adopt a multi-layer structure, including an electron/hole transport layer^38^ and an electron/hole blocking layer^39^. The addition of these structures aims to facilitate the injection of electrons and holes or to utilize energy level differences to trap electrons and holes within the emitting layer, thereby increasing the chances of radiative recombination^40,41^. In the process of making PeLED, while the mentioned measures contribute to improving the brightness and efficiency of the devices, it should be noted that adding each additional layer increases the internal resistance of the component, theoretically. This increase in internal resistance may result in some non-radiative energy loss during PeLED operation. In this work, we utilized the SSVD method to deposit CsPbBr_3_ material, simplifying the device fabrication process. Proper heat treatment processes promote the growth of the emitting layer’s crystals, improve the crystal structure, and reduce defect formation. Additionally, to minimize energy loss, the PeLED devices in the study employed a simplified structure consisting only of ITO as the cathode, CsPbBr_3_ as the emitting layer, and carbon as the anode, without the addition of the electron/hole transport layers in PeLED structures. The study demonstrated that this straightforward PeLED structure achieves high luminance at low operating voltages. This approach provides a simple, rapid, and cost-effective fabrication method for future academic research in the field of PeLED.

## Experimental section

### Materials

Acetone (ACE, 99.5%) was purchased from (LCY Chemical), Isopropyl alcohol (IPA, 99.5%) was purchased from (LCY Chemical), Conductive substrate (ITO, 25 mm × 25 mm × 7 mm, Resistor 7 ohms) was purchased from Ruilong Co., Ltd., Perovskite powder (CsPbBr_3_) were purchased from Utang Co., Ltd., Carbon glue (C, PF-407C) were purchased from Acheson. All materials and solvents were used without further purification.

### The fabrication process of PeLED

**Figure 1:**
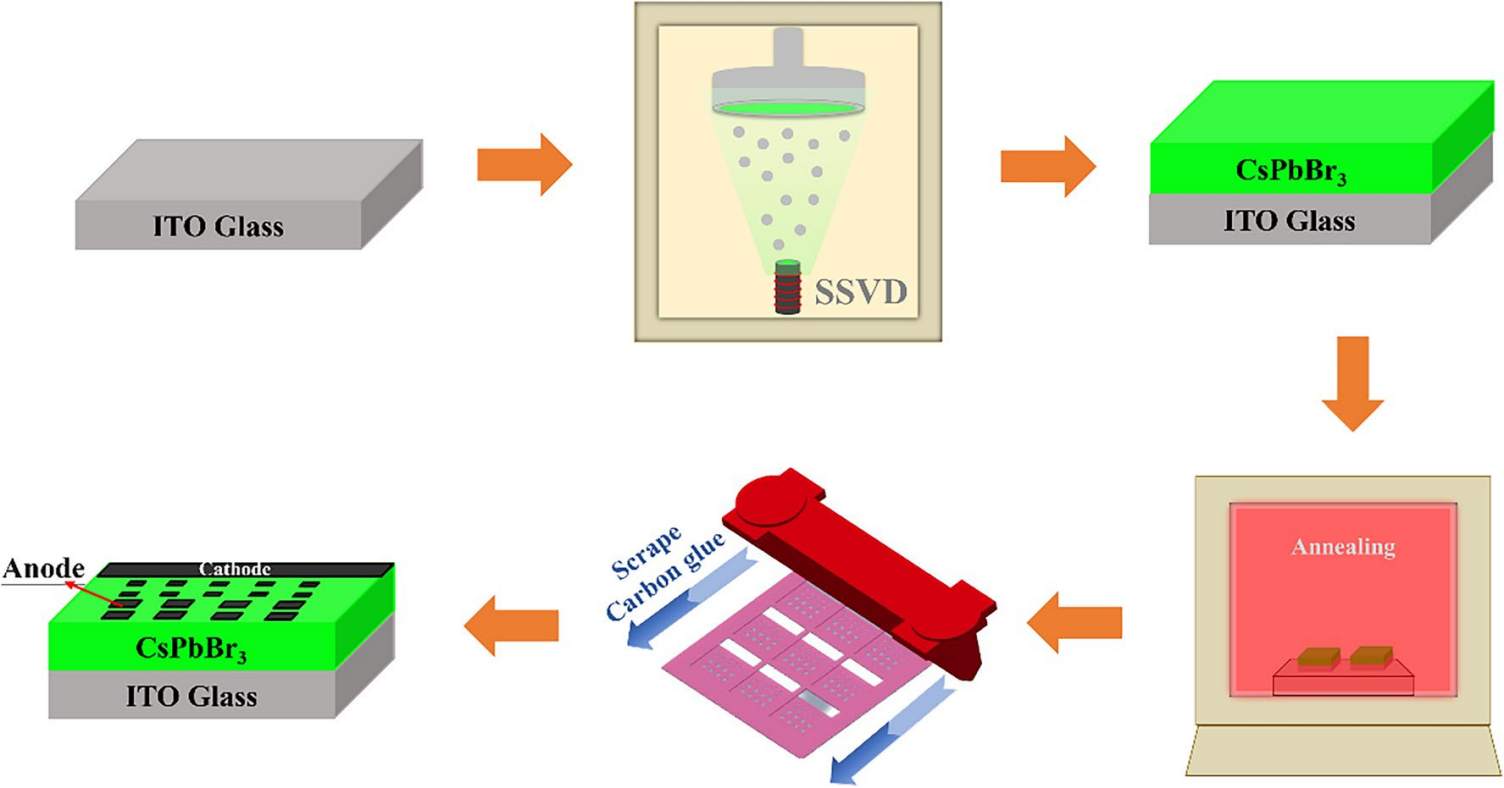
Schematic illustration of the PeLED fabrication process.

Glass and ITO substrates are sequentially immersed in a cleaning solution, followed by ACE and IPA. Ultrasonic cleaning is used to clean the glass surface, and then nitrogen gas is used to dry the substrate surface. Finally, UV-ozone exposure for 10 min is used to complete the substrate cleaning. The subsequent experimental steps are depicted in Fig. 1. The cleaned substrate is placed in the vacuum chamber of a thermal evaporation machine, achieving a vacuum level of approximately 8 × 10^−6^ torr. CsPbBr_3_ powder is placed in a molybdenum boat and is sublimated onto the substrate using resistive heating, resulting in the deposition of a CsPbBr_3_ film with a thickness of about 500 nm. After the thermal evaporation process, the sample is placed in a high-temperature furnace. Under atmospheric conditions, it is heated at a rate of 10 °C/min, and annealing is carried out at different temperatures, specifically 250 °C, 300 °C, 350 °C, and 400 °C. Finally, the substrate is placed within a mask with predefined patterns, and a uniform layer of carbon paste is applied to the substrate using a scraper. After the carbon paste is dried, the device is ready for subsequent analysis and measurements.

### Characterization

Thermal Evaporation Coater (Taiwan Instrument Research Institute). Ultraviolet/visible Spectrophotometer (UV–vis, HITACHI U-2900), Sample measurements were conducted for wavelengths ranging from 300 to 800 nm at a scanning speed of 100 nm/min. X-ray Diffractometer (XRD, Bruker D8 ADVANCE) Sample was scanned at a scanning speed of 0.05°/s over the angle range of 10° to 50°. Scanning Electron Microscope (SEM, HITACHI S-4300) sample morphology was observed at an acceleration voltage of 10.0 kV. The ultraviolet photoelectron spectrometer (UPS, Sigma Probe) sample was measured with helium excitation at 21.2 eV, and a negative bias voltage of − 5 V was applied. Integrating-sphere (Labsphere), Spectrometer (Ocean Optics USB2000+), and Power Supply (Keithley2400) Photoluminescence (PL) measurements of the thin film were conducted using a light source at 402 nm for excitation. Ultraviolet Lamp (Amalytik Jenak) thin film was excited and observed for luminescence using a light source at 365 nm. PeLED measurements were all conducted in atmospheric conditions, measuring current density–voltage-luminance (J-V-L) using a Power Supply (Keithley 2400) and a Luminance Meter (TOPCON, BM-7AC).

## Results and discussion

### Improving the quality and optical properties of CsPbBr_3_ perovskite films through the thermal annealing process

Currently, the SSVD method for preparing CsPbBr_3_ PeLED is relatively less commonly used by scientists, with fewer reported studies than the dual-source co-evaporation method. Therefore, this study aims to provide a comprehensive exploration of the potential applications of SSVD-prepared CsPbBr_3_ PeLED in LED technology. In this study, we employed CaPbBr_3_ as the source material for the SSVD process. Since our research was conducted in ambient conditions, precautions were taken to shield the CsPbBr_3_ film from environmental factors like moisture and oxygen^42,43^, which could lead to numerous pores and defects on the film surface. These issues could affect the subsequent optoelectronic performance and research outcomes. Consequently, we fabricated CsPbBr_3_ perovskite films with a thickness of approximately 500 nm for analysis, which differs from other studies that have created thinner films of less than 100 nm within gloveboxes^29^.

**Figure 2:**
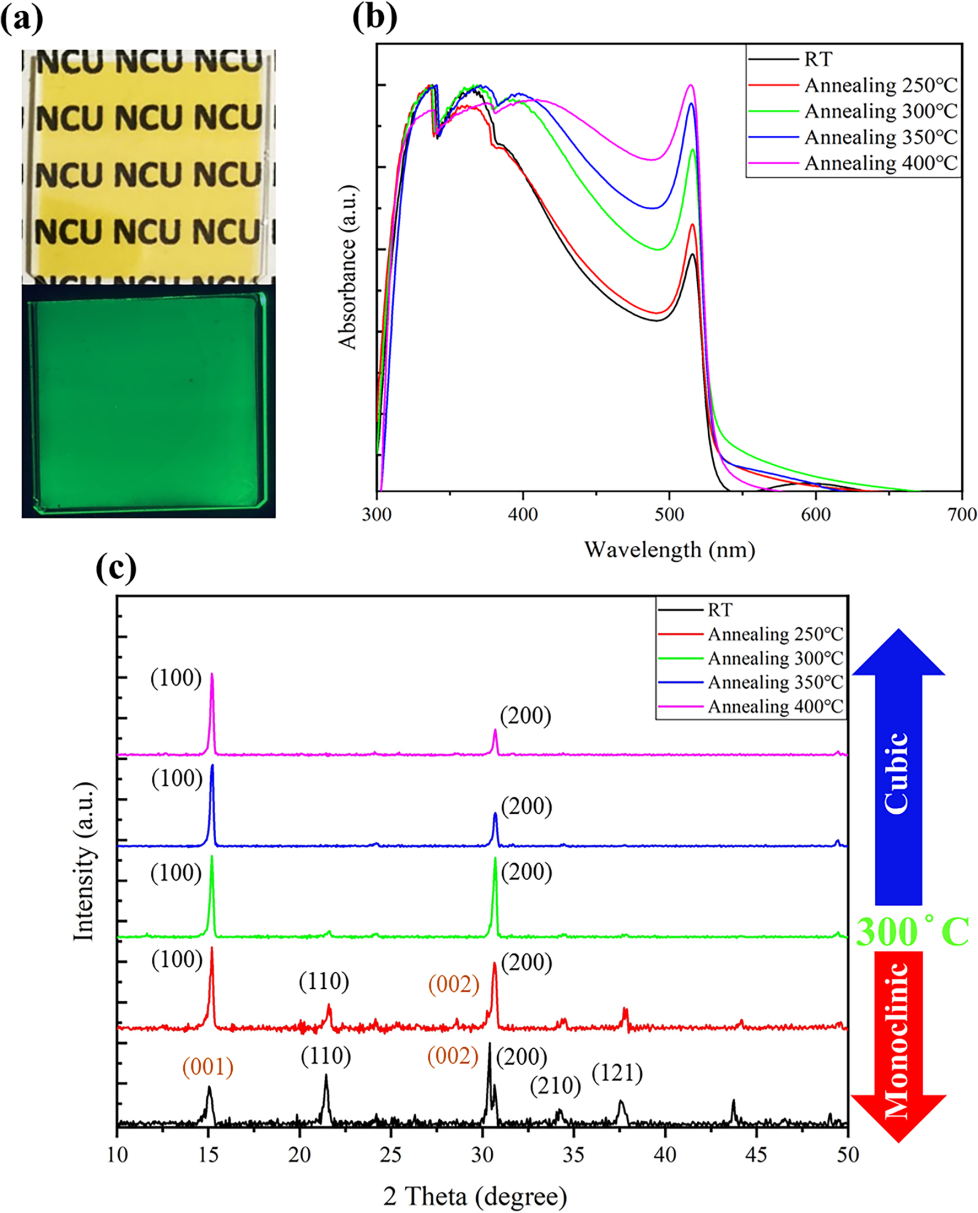
The impact of different annealing processes at various temperatures on the quality and crystalline structure of CsPbBr_3_ thin films. (**a**) The appearance of freshly deposited CsPbBr_3_ thin films and their appearance after excitation are presented. (**b**) UV–Vis measurement results demonstrate that with increasing annealing temperature, the absorption peak of CsPbBr_3_ intensifies at around 520 nm, indicative of enhanced film grain growth and improved crystal quality. (**c**) X-ray diffraction (XRD) analysis reveals that as the annealing temperature rises, a change occurs in the film’s crystal structure. Specifically, when the annealing temperature exceeds 300 °C, the crystal phase transitions from the monoclinic phase to the cubic phase.

We first conducted an initial visual analysis of the freshly deposited films (Fig. 2a). Under daylight illumination, the films appeared transparent with a yellowish hue. When exposed to UV light (source: 365 nm), the films emitted green light, confirming the photoluminescent nature of the films. However, we also observed that the brightness of the films was relatively low. This phenomenon could be attributed to the presence of defects within the film, which have a detrimental effect on the formation and recombination of electron–hole pairs. These defects can reduce the bandgap of the film, preventing some electron–hole pairs from being excited to higher energy states. Additionally, they can increase the probability of non-radiative recombination, leading to the suppression of photon emission by some electron–hole pairs. These factors collectively influence the brightness and performance of PeLED. As the quality of the perovskite emission layer is a critical parameter determining the luminous efficiency of PeLED devices, it’s important to address the issue of non-radiative recombination defects prevalent in polycrystalline perovskite thin films, including point defects and extended defects. Mitigating non-radiative recombination defects in perovskite materials is a crucial prerequisite for achieving high-performance devices in luminescent applications. Therefore, in this study, we employed a thermal annealing process to enhance film crystallinity and ameliorate non-radiative recombination issues. Thermal annealing provides sufficient thermal energy to the crystal grains, promoting their vibrational motion and diffusion while facilitating the fusion and growth of different crystal grains to form larger, high-quality crystals. In this research, we conducted thermal annealing processes at temperatures of 250 °C, 300 °C, 350 °C, and 400 °C, with a consistent annealing duration of 100 min, to examine the impact of various thermal treatment temperatures on the quality of the emission layer.

From the UV–Vis measurements of the CsPbBr_3_ emission layer (Fig. 2b), it is evident that with increasing temperature, the absorption peak at 520 nm strengthens, indicating an improvement in the film’s crystalline quality. When the thermal annealing temperature is set at 250 °C, there is no significant enhancement in the absorption peak. This may be attributed to the fact that the annealing temperature is too low to induce substantial changes in the crystal structure. However, as the annealing temperature is raised to 300 °C, there is a pronounced enhancement in the absorption peak, indicating a reduction in crystal grain defects and their reorganization. Further elevating the annealing temperature to 350 °C and 400 °C sustains the strengthening of the absorption peak at 520 nm, suggesting that the crystal grains have acquired sufficient energy to engulf smaller grains through coalescence and growth. Additionally, the enhancement of the 520 nm absorption peak is accompanied by a relative decrease in the 320 nm absorption peak. According to reported literature^37,44^, the 320 nm absorption peak may result from a reaction between small amounts of PbBr_2_ and CsPbBr_3_ during the deposition process, forming CsPb_2_Br_5_, which can lead to a reduction in PL and electroluminescence radiative recombination.

The use of thermal annealing helps improve the crystalline quality. Therefore, we employed X-ray diffraction (XRD) analysis to examine the crystalline structure of the films and investigate the effects of different annealing temperatures (Fig. 2c). At room temperature, CsPbBr_3_ exhibits a monoclinic phase (JCPDS No 00-018-0364) with characteristic peaks and corresponding crystal planes at 15.08° (001), 21.65° (110), 30.4° (002), 30.7° (200), 34.2° (210), and 37.75° (121). When annealed at 250 °C, the peak at 15.08° transitions to 15.2° (100), while the double peaks at 30.4° (002) and 30.7° (200) remain. This is possible because the lower annealing temperature doesn’t provide sufficient thermal energy to induce significant changes in the crystal structure. However, as the annealing temperature increases beyond 300 °C, the film’s crystal phase transitions from monoclinic to cubic (JCPDS No 00-054-0752). The main reason for this is that the double peaks at 30.4° (002) and 30.7° (200) merge into a single peak at 30.7° (200), and the peak at 15.08° (001) shifts to 15.2° (100). This transformation occurs because when the annealing temperature approaches or exceeds the melting point of PbBr_2_ (372 °C), Pb and Br atoms undergo perturbation, leading to the rearrangement of atoms or their departure from the surface, resulting in changes in the crystal structure and the disappearance of defects^45–47^. Furthermore, as the temperature increases, there is a gradual shift in the dominant crystal planes to (100) and (200). This indicates that raising the temperature provides sufficient thermal energy for recrystallizing the original CsPbBr_3_ film. The results of XRD and UV–Vis measurements show that, although the quality of the film improves as the annealing temperature increases, it does not necessarily mean that radiative recombination of the film is enhanced. Therefore, we conducted PL measurements to examine the influence of the temperature process on the luminescent properties of the film.

**Figure 3:**
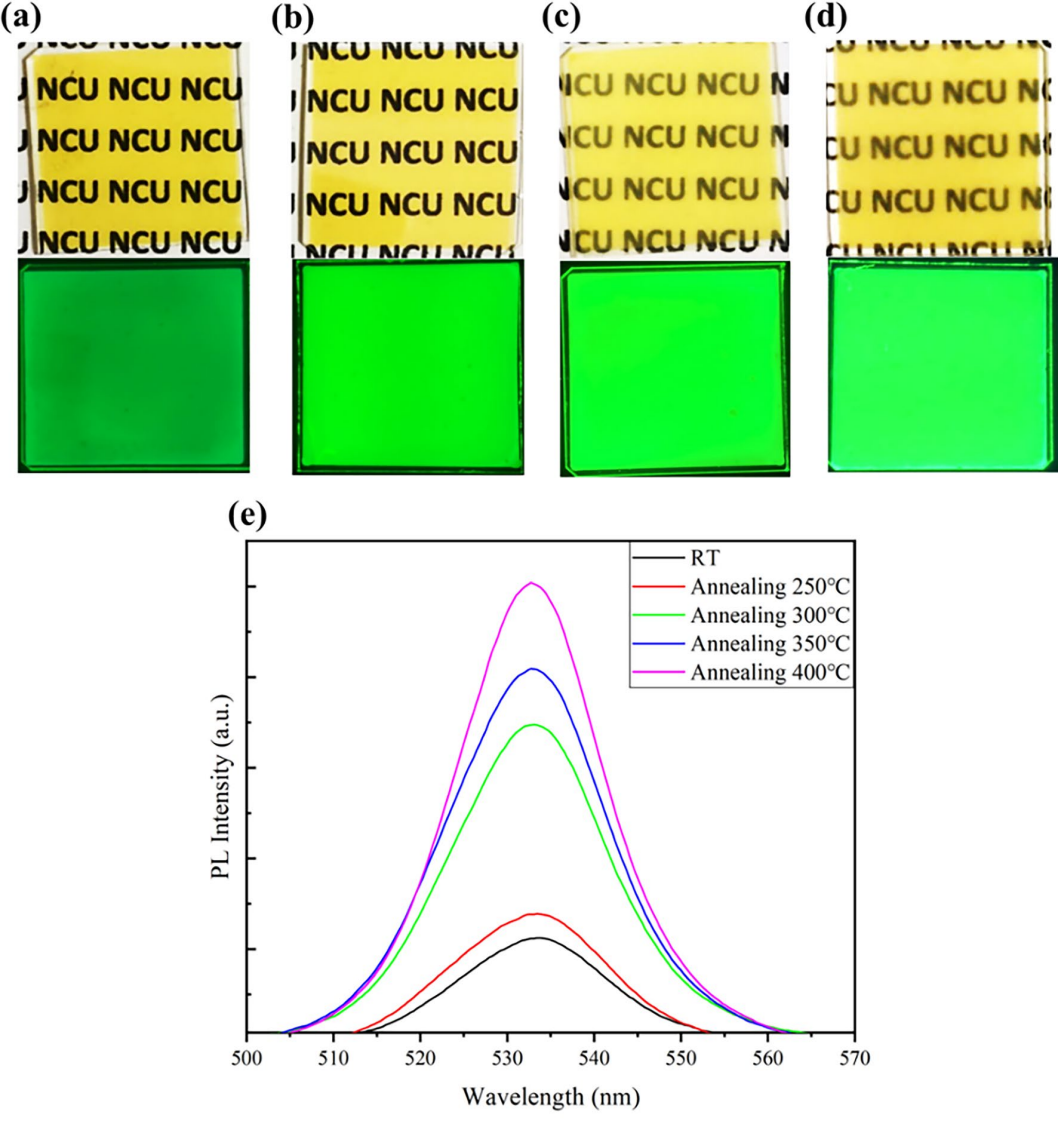
(**a**–**d**) Depicts the appearance (top) and UV-excited state (bottom) of CsPbBr_3_ thin films after different annealing treatments. Correspond to annealing temperatures of 250 °C, 300 °C, 350 °C, and 400 °C, respectively. (**e**) The PL spectra of the films after various annealing treatments. Films annealed at room temperature and 250 °C exhibit relatively weaker PL intensity, while films annealed at temperatures above 300 °C demonstrate a significant enhancement in PL intensity.

The appearance and optical properties of CsPbBr_3_ films subjected to annealing at different temperatures are shown in the figure (Fig. 3a–d). We observed that the annealing process had no significant effect on the appearance of the films, which remained predominantly light yellow. When excited by UV light, all the films emitted green light, and the luminescence intensity increased with higher annealing temperatures. To further understand the differences in PL intensity and emission wavelength among the various films, measurements were conducted using an integrating sphere system with excitation by a 405 nm light source, and the results are shown in the figure (Fig. 3e). The emission wavelength of CsPbBr_3_ films was approximately 533 nm, and the full width at half maximum (FWHM) was less than 18 nm in all cases. The annealed films did not exhibit a noticeable redshift in wavelength. According to X-ray diffraction (XRD) analysis, it is known that CsPbBr_3_ can exist in two crystal phases, monoclinic and cubic. The films annealed at room temperature, and 250 °C predominantly exhibited the monoclinic phase, while those annealed at temperatures exceeding 300 °C underwent a phase transition to the cubic phase^47^. This transition in crystal phases was also reflected in the PL spectra, with films annealed at temperatures above 300 °C showing a significant enhancement in PL intensity. This enhancement could be attributed to the differing influence of the crystal phases on electron–hole recombination or the improved crystal quality resulting from the dominance of specific crystal facets in the annealed films, thereby contributing to enhanced radiative recombination effects and reduced defect presence^45,48^. However, when the annealing temperature reached higher levels of 350 °C and 400 °C, the appearance of the films became somewhat hazy and opaque. This haziness was particularly pronounced at 400 °C. As speculated earlier, the films’ appearance is hazy, which may be due to their poor density. This could be because the temperature of 400 °C exceeds the melting point of PbBr_2_, causing some PbBr_2_ within CsPbBr_3_ to separate. This affects grain growth, and the separated PbBr_2_ may subsequently form voids, affecting the performance and efficiency of PeLED.

### Analysis of CsPbBr_3_ thin film morphology via the thermal annealing process

**Figure 4:**
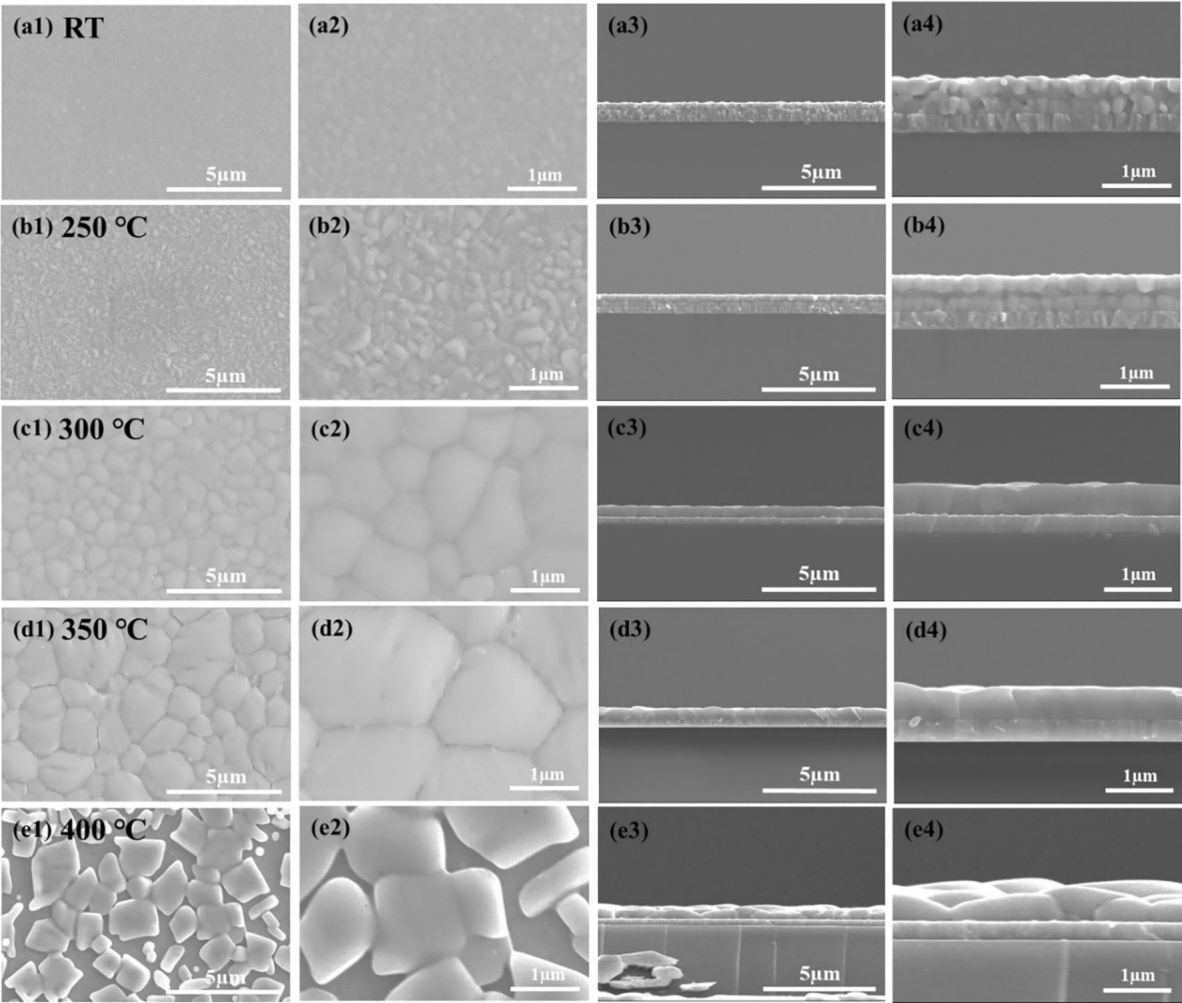
There are significant variations in grain size and compactness of CsPbBr_3_ thin films at different annealing temperatures. (**a**–**e**) Correspond to room temperature, 250 °C, 300 °C, 350 °C, and 400 °C, respectively.

The CsPbBr_3_ film was deposited on an ITO substrate, and the films were observed by SEM at room temperature as well as after annealing at 250 °C, 300 °C, 350 °C, and 400 °C, as shown in Fig. 4. When observing the film at room temperature from the top view, it appeared to have a relatively high density (Fig. 4a1,2), but the grain size was only about 100 nm. However, from the side view, it was evident that the grains were stacked chaotically, and there was a generation of pinholes (Fig. 4a3,4). This condition can lead to non-radiative recombination in the film, subsequently affecting the luminescent performance. The film annealed at 250 °C showed grain growth in the top view (Fig. 4b1,2), but it still exhibited a stacked configuration in the side view (Fig. 4b3,4). This aligns with our previous speculation that the annealing temperature was not high enough to induce changes in the crystal structure of all grains. When the annealing temperature was raised to 300 °C and 350 °C, small grains were able to merge with each other due to the thermal energy provided, resulting in grain sizes of 1–2 µm (Fig. 4c1,2,d1,2). At this point, the film was very dense, and no pinholes were observed. The stacking of grains transformed into neatly arranged individual grains, and no pores were observed at both high and low magnifications (Fig. 4c3,4,d3,4). The SEM results confirmed the enhancement of the UV–Vis absorption peaks, the transition to the cubic phase in XRD, and a significant increase in PL intensity. These results demonstrate the critical influence of the quantity of grain boundaries on the film’s quality and radiative recombination efficiency^46,49^. The more grain boundaries there are, the more defects the material will generate, resulting in poor film quality and inadequate PL intensity. However, when the annealing temperature reached 400 °C, numerous pores began to appear, and the grain sizes were uneven (Fig. 4e1,2). Furthermore, the film’s flatness was poor (Fig. 4e3,4). Although the CsPbBr_3_ film at 400 °C exhibited high performance in UV–Vis, XRD, and PL measurements, the formation of pores may be due to exceeding the melting point of PbBr_2_ within CsPbBr_3_, causing some PbBr_2_ to leave the film, forming products CsBr, Cs_4_PbBr_6_, and PbBr_2_. Since CsBr is highly sensitive to moisture and easily absorbs moisture, it can form numerous pores once the annealing process ends and it comes into contact with atmospheric moisture. Additionally, applying the parameters of 400 °C to PeLED may lead to leakage or efficiency issues. Based on the results of the current measurement, we selected 350 °C as the annealing temperature for the CsPbBr_3_ film in PeLED. Therefore, we used this parameter for UPS calculations.

**Figure 5:**
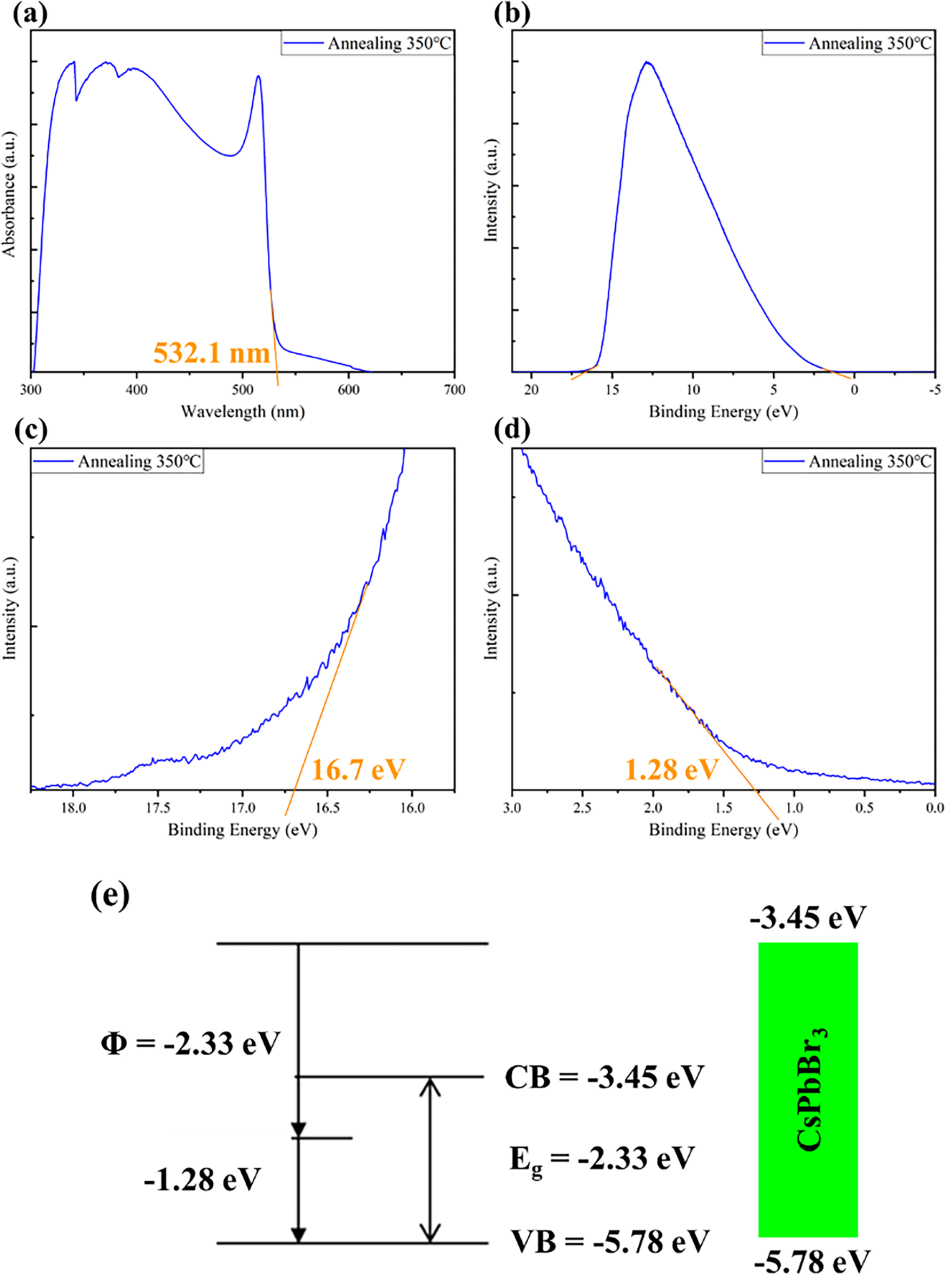
The analysis of VBM and CBM for the CsPbBr_3_ thin film subjected to a 350 °C annealing process. (**a**) UV–vis measurement: The region where the film exhibits significant absorption is at 532.1 nm to calculate the energy bandgap of CsPbBr_3_ as 2.33 eV. (**b**–**d**) UPS measurement analysis results for the CsPbBr_3_ thin film. (**e**) Schematic representation of CsPbBr_3_ energy levels. Based on the above data, the calculated energy levels for CsPbBr_3_ are VBM = − 5.78 eV and CBM = − 3.45 eV.

In order to apply the CsPbBr_3_ thin films prepared in the PeLED, it is essential to determine their Work Function (Φ). To achieve this, we utilized Ultraviolet Photoelectron Spectroscopy (UPS) to measure the energy levels of the emitting layer. The analytical results are depicted in Fig. 5. To calculate the work function, we utilized the difference between the cut-off energy (E_cut off_) of the secondary electrons and the photon energy (hν) using the following formula (1). Helium was the excitation source, producing 21.2 eV. The cut-off energy of the E_cut off_ was determined from the curve. The subsequent step involved identifying the Valence Band Maximum (VBM) of the material. This can be calculated using formula (2), where E_v_ can be determined using an extrapolation method from Fig. 5b. The final step was to determine the Conduction Band Minimum (CBM) of the material. We employed formula (3), incorporating the energy bandgap (E_g_) obtained from the UV–vis spectrum analysis to obtain the complete energy level result.

1 $$\Phi  = {\text{h}}\upnu  - {\text{E}}_{{{\text{cut off}}}} $$

2 $${\text{VBM}} = \Phi + {\text{ E}}_{{\text{v}}}$$

3 $${\text{CBM}} = {\text{VBM}} + {\text{ E}}_{{\text{g}}}$$

By plugging in the values obtained from the UV–vis and UPS measurements into the formulas above, we determined that the energy levels of CsPbBr_3_ were CBM = − 3.45 eV and VBM = − 5.78 eV, as shown in Fig. 5e. The calculated results align well with the existing literature^50,51^. This information provides a deeper understanding of the energy levels of CsPbBr_3_, laying the foundation for its application as the emitting layer in PeLED.

### Analysis of the impact of the thermal annealing process on CsPbBr_3_ PeLED device performance

**Figure 6:**
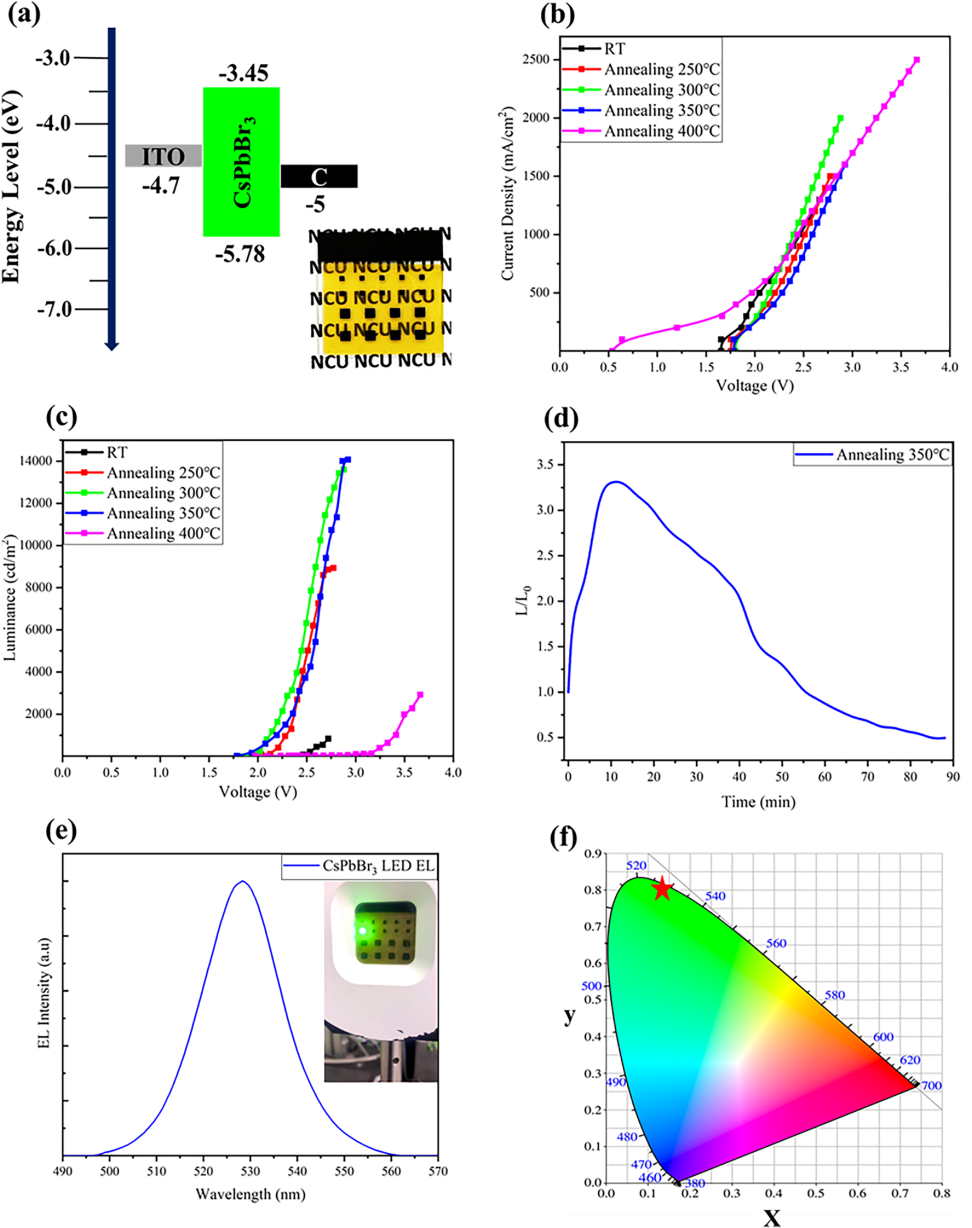
(**a**) Appearance and structural energy diagram of the PeLED. (**b**) PeLED current density–voltage (J–V) curves. (**c**) PeLED luminance–voltage (L–V) curves. (**d**) The operational stability test of the devices annealed at 350 °C, measured under atmospheric conditions with an initial brightness of 100 cd/m^2^. (**e**) The emission wavelength of the device is 528 nm. Inset: a photograph of the working PeLED. (**f**) The device’s coordinates are in the CIE chromaticity diagram (0.1475, 0.8045).

We have fabricated a simple structure of PeLED, as shown in Fig. 6a. This structure includes an ITO anode (250 nm), a CsPbBr_3_ emitting layer (approximately 500 nm), and a carbon cathode (about 30 µm). Notably, our PeLED device is without any electron/hole transport layers. Despite a relatively poor energy level matching, this demonstrates the high quality and low defect of the emitting layer in our PeLED. Due to the absence of electron/hole transport layers, the initial voltage of the device is approximately 1.7 V to 1.8 V (Fig. 6b), close to the threshold in CsPbBr_3_ LED theory and similar to previous research literature^52^. However, the device subjected to 400 °C annealing exhibited a large number of holes in the light-emitting layer during the annealing process, which caused leakage current problems in the components.

The brightness-voltage characteristics of all devices are displayed in Fig. 6c. Firstly, for the non-annealed PeLED, a maximum luminance of 838.8 cd/m^2^ is achieved at 2.72 V. Although the device can emit light, its brightness is significantly lower. This is due to the smaller grain size and stacking of the emitting layer, which results in an increased number of grain boundaries. Grain boundaries can easily hinder the transfer of electrons and holes, causing part of the charges to be quenched before recombination, generating excess heat energy and reducing device efficiency. Furthermore, when the emitting layer generates photons, some of these photons can be reabsorbed by other grains, causing energy loss and affecting PeLED brightness. For a device annealed at 250 °C, the maximum brightness is 8,934 cd/m^2^ at 2.77 V. Although the annealing temperature is relatively lower, it can still improve the quality of part of the light-emitting layer, and the brightness is ten times higher than that of PeLED without heat treatment. The CsPbBr_3_ emitting layer annealed at 350 °C exhibits excellent performance in various measurements. After device fabrication, this PeLED reaches a maximum luminance of 14,079 cd/m^2^ at 2.92 V. This indicates that the annealed emitting layer has fewer grain boundaries and larger grain sizes, reducing defects and promoting electron–hole recombination. This results in a significant increase in brightness, with a gain of 16.8 times compared to the non-annealed PeLED. It is worth noting that although the PL of the light-emitting layer exhibits more vigorous luminous intensity after annealing at 400 °C, the prepared PeLED device exhibits relatively low brightness. The main reason for this phenomenon is that a large number of holes are formed during the annealing process, which leads to significant leakage current during testing and thus affects the luminous efficiency of PeLED. This causes the device to start at a relatively lower initial voltage of about 0.5 V (Fig. 6b), which is unfavorable for device performance. That means that to make the PeLED work, more energy needs to be input, which is not efficiently converted into brightness, resulting in lower PeLED efficiency. In summary, the thermal annealing process has a positive impact on the performance of CsPbBr_3_ PeLED devices, improving their luminous efficiency. However, it is essential to carefully control the annealing temperature to avoid issues such as void formation and other adverse effects.

In order to evaluate the operational stability of CsPbBr_3_ PeLED, we conducted the test in ambient conditions with a fixed operating voltage of 2 V and an initial brightness of 100 cd/m^2^. After the PeLED device started operating, the brightness increased rapidly but later exhibited a gradual decline, ultimately reaching the T_50_ lifetime of 88 min (Fig. 6d). The experimental results indicate that, although PeLED can sustain operation for a while, the brightness of the device shows an initial increasing and then decreasing trend. According to the previous study^53^, this phenomenon is attributed to the presence of ion migration within the device. Therefore, there is still room for further improvement in improving the light-emitting layer. Additionally, the emitted wavelength of the CsPbBr_3_ PeLED device we prepared is 528 nm (Fig. 6e), with CIE coordinates of (0.1475, 0.8045) (Fig. 6f). These coordinates are very close to the color purity edge, indicating that CsPbBr_3_ PeLED exhibits superior color purity compared to green OLED. Based on the research results, the CsPbBr_3_ emitting layer annealed at 350 °C demonstrates the best performance in PeLED devices. Furthermore, the successfully prepared CsPbBr_3_ PeLED device operates at a driving voltage of less than 3 V when reaching its maximum luminance (Table 1). This is a significant improvement in the operating voltage required for maximum brightness compared to other reported devices. However, we acknowledge that the current efficiency is relatively lower, primarily due to the lack of electron/hole transport layers in our device structure. According to various studies, the inclusion of electron/hole transport layers in PeLED devices can further enhance the injection efficiency of electrons and holes^54–56^, ultimately improving radiative recombination in the emitting layer and potentially leading to higher device brightness and current efficiency. Based on the results presented here, annealing can effectively enhance the optoelectronic performance of CsPbBr_3_ films. Future research can focus on optimizing device structures and material properties to achieve higher efficiency and more stable CsPbBr_3_ PeLED.

**Table 1:** The photovoltaic efficiency of CsPbBr_3_ PeLED annealed at different temperatures.

Device	Luminance max (cd/m^2^)	Voltage (V)	Current efficiency (cd/A)
RT	838.8	2.72	0.06
250 °C	8934	2.77	0.60
300 °C	13,600	2.88	0.68
350 °C	14,079	2.92	0.93
400 °C	2920	3.66	0.12

## Conclusion

This work utilized CsPbBr_3_ as the raw material and employed the SSVD process to deposit CsPbBr_3_ thin films. However, the initially deposited films exhibited characteristics of grain stacking and a higher number of grain boundaries, leading to an increased presence of defects in the film, consequently resulting in relatively lower PL intensity. These defects adversely affected the radiative recombination of electrons and holes. Thermal annealing was employed to enhance the crystalline quality of the films to address this non-radiative recombination issue. Through an appropriate thermal annealing process, the crystalline quality of CsPbBr_3_ films was improved, leading to increased grain size and reduced grain boundaries, ultimately enhancing lattice integrity and minimizing the presence of defects. As a result of thermal annealing, the crystal phase of CsPbBr_3_ films transitioned from the monoclinic to the cubic. Furthermore, the films exhibited a significant increase in PL intensity and enhanced absorption at 520 nm, indicating improved radiative recombination and, consequently, improved light-emitting performance. Additionally, we successfully fabricated simple-structured CsPbBr_3_ PeLED devices using thermally annealed films. In these devices, no electron/hole transport layers were added. Specifically, after thermal annealing at 350 °C, these devices achieved a maximum luminance of 14,079 cd/m^2^ at a driving voltage of 2.92 V. Their CIE coordinates were (0.1475, 0.8045), indicating a significant improvement in luminance compared to the untreated devices, which exhibited 16.8 times higher maximum luminance. However, it’s worth noting that these devices are without electron and hole transport layers, leading to a requirement for higher current density, which, in turn, resulted in relatively lower device performance. Future research efforts may optimize the device structure and material properties to achieve higher efficiency and stability in CsPbBr_3_ PeLED. The findings of this study suggest that the SSVD process for CsPbBr_3_ PeLED fabrication holds promising applications, providing an alternative option in display technology.

## Data Availability

The datasets used and/or analyzed during the current study are available from the corresponding author on reasonable request.
